# RNA-Binding Proteins in the Regulation of miRNA Activity: A Focus on Neuronal Functions

**DOI:** 10.3390/biom5042363

**Published:** 2015-09-30

**Authors:** Alessia Loffreda, Aurora Rigamonti, Silvia M. L. Barabino, Silvia C. Lenzken

**Affiliations:** Department of Biotechnology and Biosciences, University of Milano-Bicocca, Piazza della Scienza 2, 20126 Milan, Italy; E-Mails: a.loffreda@campus.unimib.it (A.L.); a.rigamonti8@campus.unimib.it (A.R.); silvia.barabino@unimib.it (S.M.L.B.)

**Keywords:** miRNA, RNA binding proteins, RBPs, neuronal-gene-expression-regulation, miRNA-RBP cooperation, miRNA-RBP competition

## Abstract

Posttranscriptional modifications of messenger RNAs (mRNAs) are key processes in the fine-tuning of cellular homeostasis. Two major actors in this scenario are RNA binding proteins (RBPs) and microRNAs (miRNAs) that together play important roles in the biogenesis, turnover, translation and localization of mRNAs. This review will highlight recent advances in the understanding of the role of RBPs in the regulation of the maturation and the function of miRNAs. The interplay between miRNAs and RBPs is discussed specifically in the context of neuronal development and function.

## 1. Introduction

Let-7, the first phylogenetically conserved miRNA, was identified in *C. elegans* in 2000 [1]. Since then, thousands of miRNA have been identified in eukaryotes. According to the 21st release of miRBase in 2014, the human genome codes for 1881 precursors and for 2588 mature miRNAs [2]. Since miRNAs are involved in multiple functions related to the expression of genes, it is not surprising that their deregulation is linked to human disease.

Here, we review recent literature that highlights the role of RNA-binding proteins (RBPs) in the regulation of miRNA biogenesis and function with a special focus on their involvement in neuronal development and function.

### 1.1. MiRNA Biogenesis

MiRNAs are either encoded by specific genes that can be organized in clusters or can be embedded in protein-coding genes (for a recent review on miRNA transcription see [3]). RNA polymerase II (RNAPol II) transcribes the majority of miRNA-encoding genes to generate a long, highly structured primary miRNA (pri-miRNA) molecule. In addition to RNA Pol II also RNAPol III has been reported to transcribe miRNAs but only on few selected loci (the chr19 miRNA cluster (C19MC) [4], *SNAR-A* (also known as *CBL-1* [5]) and *MIR886* [6]). However, a recent study that characterized the RNAPol III-occupied loci by chromatin immunoprecipitation (ChIP) confirmed enrichment on SNAR-A and MIR886 but not on C19CM [7].

**Figure 1:**
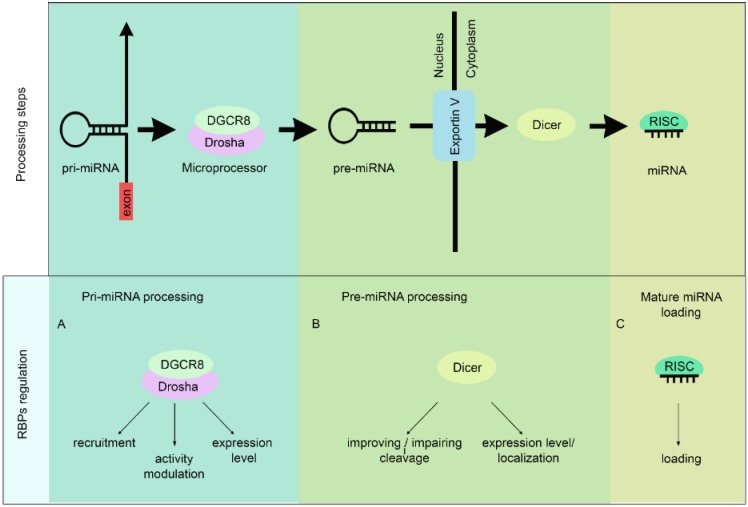
MiRNA biogenesis and RBPs regulation. Upper panel: schematic representation of miRNA biogenesis. The initiation step is mediated by the Drosha–DGCR8 complex (also known as the Microprocessor complex). This complex generates the pri-miRNA, which is recognized and exported by the nuclear export factor Exportin 5. In the cytoplasm, the RNase III Dicer catalysis the second processing step generating miRNA duplexes. While one strand of the duplex is loaded on the RISC, the other strand is degraded. Lower panel: Processing steps in which the RBPs could regulate miRNA biogenesis.

Depending on how the pri-miRNA transcript is processed, miRNAs can be divided into two classes (for detailed reviews of miRNA processing see [8,9]). In the case of the canonical miRNAs, the Microprocessor complex that includes the RNAseIII Drosha and the double-stranded RNA-binding protein DGCR8 (DiGeorge syndrome critical region 8 gene) cleaves the pri-miRNA to generate a 60–70 nt long hairpin precursor miRNA (pre-miRNA). Exportin V then exports the pre-miRNA to the cytoplasm where the RNAse Dicer cleaves it, giving rise to the mature miRNA (see Figure 1, upper panel). In contrast, non-canonical miRNAs do not require the Microprocessor complex for their processing. For example, intron-encoded miRNAs (mirtrons) are excised during the splicing reaction by the spliceosome and are direct Dicer substrates [10].

### 1.2. RNA Modifications in miRNA Biogenesis

After transcription, RNA molecules undergo extensive chemical modifications that can influence their stability, localization and function. Particularly, ribosomal RNAs and tRNAs are extensively modified. Modifications can occur on the bases as well as on the ribose. To date, 109 nucleoside modifications are listed in The RNA Modification Database (RNAMDB, http://mods.rna.albany.edu/), many of which are conserved throughout bacteria, archaea, and eukaryotes. Recently, several RNA modifications have been found also in regulatory RNAs. For example, the N(6)-methyl-adenosine (m(6)A), the most abundant modification in eukaryotic mRNAs, alters the structure of both mRNAs and long non-coding RNAs, thereby affecting their RNA–protein interactions [11]. Recently, Alarçon and colleagues discovered that m(6)A is a key mark that promotes pri-miRNA processing by the Microprocessor complex [12]. Similarly, the conversion of adenosine to inosine (A to I editing), a process mediated by adenine deaminases, was found in several miRNAs and is believed to be crucial for the regulation of their biogenesis [13]. In addition, A to I editing in the seed region of the miRNA was shown to regulate mRNA target selection and silencing efficiency [14]. Now, a very recent Clip-seq analysis reveals that Adenosine Deaminases Acting on RNA 1 (ADAR1) binds 3'UTR of nascent transcripts as well as pri-miRNAs. ADAR1 competes with factors involved in cleavage and polyadenylation causing 3'UTR lengthening. Similarly, ADAR1 was found to interact with Drosha and DGCR8 enhancing miRNA processing [15].

### 1.3. MiRNA Function

MiRNAs exert their repressive function on gene expression by regulating translation and degradation of mRNA targets. The short double-stranded miRNA generated by Dicer is bound by Argonaut proteins (Ago 1–4 in humans) in the RNA-induced silencing complex (RISC). Subsequently, the miRNA duplex is unwound and the passenger strand is removed. This step generates the mature effector RISC complex that associates with the target mRNA to induce translational repression and degradation.

In addition to the Ago proteins, the RISC complex also includes the GW-containing protein GW182 (trinucleotide repeat containing 6A, TNRC6A), which has a pivotal role in promoting target silencing. GW182 interacts with the cytoplasmic poly(A)-binding protein (PABP) [16] and recruits the CCR4-NOT [17] and PAN2-PAN3 [18] complexes to promote deadenylation, decapping and finally degradation of the mRNA. The CCR4-NOT complex also recruits the RNA helicase DDX6 that acts as a translational repressor and a decapping activator [19,20,21].

The events leading to translational repression by miRNAs are less well understood. Initiation of translation requires the formation of a 43S pre-initiation complex, consisting of eIF2-Met-tRNA-GTP, eIF1, eIF1A, eIF3, eIF5, and the 40S subunit. The recruitment of the 40S subunit is mediated by the eIF4F complex, consisting of eIF4A, eIF4G, and eIF4E [22].

Recent work has pointed to eIF4A as a crucial factor that mediates miRNA-induced translational repression even if the molecular details of this function are still controversial. eIF4A is a member of the DEA(D/H)-box RNA helicase family that probably unwinds secondary structures in the 5' untranslated region (5'UTR), thereby facilitating scanning of the 40S subunit for the initiation codon [23]. Using a cell-free system from *Drosophila* S2 cells, Fukaya *et al.* examined the association of translation initiation factors with a *Renilla* luciferase reporter for let-7 miRNA [24]. In this work, the authors showed that Ago1-RISC can repress translation by inducing the dissociation of eIF4A from the target mRNA. Moreover, they observed that direct tethering of GW182 promotes dissociation of both eIF4E and eIF4A. Thus, these authors propose that miRNAs act to block the assembly of the eIF4F complex during translation initiation [24].

Mammalian and plant cells express three eIF4A proteins (eIF4A-1–3). EIF4A1 and eIF4A2 are highly related and have been considered until recently to be functionally interchangeable (for review see [23]). However, Meijer and co-workers recently found that eIF4A2 is the only component of eIF4F required for miRNA-mediated repression [25]. Indeed, rescue experiments showed that after depletion of eIF4A2, only eIF4A2 and not eIF4A1 could restore repression [25]. Moreover, Meijer and colleagues found that eIF4A2 specifically associates with CNOT7, a component of the CCR4-NOT complex. Based on these results, the authors propose that the specific recruitment of eIF4A2 on a repressed mRNA would preclude the progression of initiation directed by eIF4G and eIF4A1.

More recently, Fukao and co-workers used a biochemical assay to monitor eIF4F recruitment in HEK293T cells [26]. They observed that association of both eIF4A1 and eIF4A2 with the reporter mRNA was reduced in the presence of miRNAs. Moreover, they found that HuD, which was previously found to interact with eIF4F and to accelerate cap-dependent translation [27], could reduce miRNA-mediated repression by preventing dissociation of eIF4A1 and eIF4A2 from the target mRNA [26]. Thus, in contrast to Meijer *et al.* [25] who proposed that eIF4A1 and eIF4A2 have opposite effects on translation with eIF4A2 repressing rather than promoting translation, Fukao *et al.* [26] proposed that both eIF4A isoforms act to stimulate cap-dependent translation.

Although it is generally accepted that miRNA-mediated silencing is due to both translational repression and mRNA degradation, the relative contribution of each mechanism is still debated. Using a reporter mRNA in a cell-free system derived from *Drosophila* S2 cells, Fukaya *et al.* demonstrated that the miRNA-RISC complex represses translation independently of deadenylation [28]. *In vitro*, this effect is independent of GW182 and does not require the CCR4-NOT complex [29]. Another study that examined the effect of miR-430 on endogenous targets in pre-gastrulation zebrafish embryos revealed that the initial translational repression can occur before complete deadenylation and mRNA decay [30]. While these studies suggest that miRNAs can affect translation efficiency independently from mRNA degradation, large-scale studies of miRNA-dependent changes in translation efficiency and in mRNA levels in two human cell lines and one primary cell type found that mRNA degradation is the dominant effect of miRNAs-induced silencing in mammals [31,32]. More recently, a high-throughput study measured the poly(A) tail length of individual mRNAs isolated from yeast, plant, fly, and mammalian cells as well zebrafish and frog embryos, and then coupled ribosome profiling and RNA-seq to measure translational efficiencies in the same samples [33]. This study revealed that in early embryos (cleavage and blastula stages), translational efficiency strongly correlates with mean poly(A) tail length. After gastrulation, however, this coupling disappears. In the light of this observation, the authors propose an alternative interpretation of the effect of miR-430 expression in pre-gastrulation embryos: miRNAs promote shortening of the poly(A) tail through the recruitment of the CCR4-NOT and deadenylases. However, because of the change in translational control as well as in the stability of mRNAs with short poly(A) tails that occurs in gastrulating embryos, this shortening has different outcomes. Before gastrulation, shortening of the poly(A) tail predominantly decreases translation efficiency, while after gastrulation, deadenylation predominantly leads to mRNA degradation.

To further expand the characterization of the mechanism of the silencing effect of miRNAs *in vivo*, Eichhorn *et al.* performed ribosome footprint profiling and RNAseq to measure translational efficiencies in different primary cell types (mouse liver, macrophages and activated and non-activated B cells) and two cell lines (U2OS and NIH3T3 cells) in the presence or in the absence of specific miRNAs [34]. The results indicate that miRNAs predominantly induce target mRNA decay without significantly affecting translation efficiency [34].

What is the effect of miRNA-mediated gene silencing on a global scale? Schmiedel *et al.* used a combination of mathematical modelling and experimental approaches to answer this question [35]. The authors initially quantified protein levels and fluctuations by measuring single cell fluorescence of mouse embryonic stem cells (mESCs) transiently transfected with a fluorescent reporter with miRNA binding sites in the 3'UTR. Next, they built a mathematical model to distinguish between intrinsic noise, that arise from the stochasticity biogenesis and decay of the mRNA, and extrinsic noise, that arises both from external events that impact on gene expression, and the variability of the miRNA pool. The results of these combined approaches suggested that miRNAs reduce intrinsic noise in protein expression. RNAseq analysis of the transiently-transfected mESCs confirmed that miRNAs reduce total noise also for the endogenous 3'UTRs. In addition, the authors reanalyzed the microarray expression data of Dicer-deficient mESC generated by Leung *et al.* [36] and found that hundreds of genes are repressed more than twofold by the combinatorial action of miRNAs. Interestingly, most of the highly repressed genes have low expression levels so that the authors conclude that the combinatorial miRNA regulation enhances overall noise reduction by providing strong repression [35].

### 1.4. The miRNA Regulatory Network in the Neuronal Context and Its Alteration in Neurodegeneration

Recent studies in several model organisms demonstrate that miRNAs play critical roles in neurogenesis and in neuronal function (for extensive reviews see [37,38]). An example is provided by miR-125b that can regulate dendritic arborization and spontaneous neuronal activity [39]. Indeed, it has been shown that while overexpression of miR-125b in hippocampal neurons modulates dendritic spine morphology, depletion of the endogenous miR-125b using a miRNA sponge induces pruning of the dendritic arbor. The importance of miRNAs in the neuronal context is well exemplified also by miR-9. A large scale analysis of miRNAs expression revealed that miR-9 is highly enriched in both the developing and mature nervous system of vertebrates. Functional studies have highlighted a prominent role for miR-9 in regulating the behavior of neural progenitors, as well as the differentiation of some neuronal populations (for review see [40]). The expression of miR-9/9* in human fibroblasts, in synergy with miR-124, is sufficient to convert them into neurons, placing miR-9/9* at the core of the gene network controlling the neural fate [41].

While the physiological role of miRNAs in neuronal development and plasticity is well described by numerous reports, the link between miRNAs and neurodegeneration is mainly supported by conditional Dicer knockouts studies [42,43,44,45]. Indeed, in various model organisms, Dicer deficiency (that leads to a global disruption of miRNA biogenesis) results in neurodegenerative features. For example, the loss of miRNAs in mouse dopaminergic neurons leads to reduced locomotion, which is reminiscent of the phenotype of Parkinson’s Disease [46]. Alterations in dendritic spine density and length have been observed in the hippocampus of Dicer-deficient mice, which could have profound effects on memory. Similar deficits are associated with neurological disorders such as Alzheimer Disease, schizophrenia and Fragile X mental retardation [42]. Dicer1 deletion in spinal motor neurons results in a spinal muscular atrophy (SMA)-like phenotype [47].

Taken together these and other reports that we cannot mention in this review due to length restrictions indicated that alterations in the miRNA repertoire may be the cause of or may contribute to the etiology of neurological disorders [48,49]. In fact, in less than 10 years since the first Dicer knockout study [50], several authors have reported the misregulation of specific miRNAs in mice models of different neurodegenerative disorders, which in some instances were confirmed in *post mortem* samples of the affected tissues (miR-206/miR-153 in Alzheimer’s disease [51,52]; miR-34b/miR-9/miR-9* in Huntington’s disease [53,54]; miR-9 in SMA [47]); miR-206/miR-338-3p/miR-451 in ALS [55,56]. However, the pathological contribution of the individual miRNAs to each of these diseases is still under investigation.

## 2. RBPs Regulate miRNAs Biogenesis

MiRNAs biogenesis is a highly regulated process. Due to their intrinsic ability to bind nucleic acids, RBPs are involved in almost every step of miRNA biogenesis, from primary miRNA processing to RISC formation (for an extensive review of regulatory mechanisms of miRNA biogenesis see [57]). Here, we will mainly focus on examples of RBPs that participate in miRNA biogenesis in the framework of neuronal function (Table 1).

**Table 1:** RBPs affecting miRNA biogenesis.

RBP	Main RBPs Functions	miRNA Target	Affected Neuronal Functions	References *
TDP-43 **	Transcriptional regulation, alternative splicing, mRNA transport and translation	miR-132	Neuronal plasticity, synapse formation, neurite outgrowth [39,58,59]	[60]
FUS **		miR-9; miR-125; miR-132; miR-200a; miR-141	Synapse formation, neuronal plasticity, neurite outgrowth, neuronal differentiation and proliferation [39,53,58,59,61]	[62,63]
TAF15	Transcriptional regulation	miR-17	Neuronal proliferation and survival [64]	[65]
Lin-28	mRNA processing and translation	let-7	Neuronal stem-cell commitment, neuronal proliferation, tissue-regeneration [66,67,68,69,70]	[67,71,72,73]
NF45/NF90	mRNA transport/ stability	let-7	*See above for let-7*	[74]
DDX6	mRNA translation and degradation	let-7	*See above for let-7*	[75]
hnRNP A1	mRNA splicing and transport	miR 18a; let 7	Neuronal survival and proliferation [64] *See above for let-7*	[76,77]
MSI2/HuR	mRNA stability and localization	miR-7	Synuclein levels regulation, neurite outgrowth [78,79]	[80]
DHX36	mRNA stability	miR-134	Neuronal plasticity [59,81,82]	[59]

* articles where RBP-miRNA regulation was reported. ** both TDP43 and FUS have been implicated in the same cellular functions.

### 2.1. Pri-miRNA Processing

RBPs can influence Drosha-mediated pri-miRNA processing by acting at three levels (Figure 1A). First, RBPs can promote Drosha recruitment to pri-miRNA transcription sites. Second, they can enhance or impair Drosha cleavage ability. It should be mentioned in this regard that these two mechanisms might take place simultaneously. Finally, RBPs can regulate the expression level of the Microprocessor complex.

#### 2.1.1. Drosha Recruitment to Pri-miRNA Transcription Sites

The DNA/RNA-binding protein FUS (Fused in Sarcome/Translocated in Sarcoma) has been involved in several biological processes such as transcription regulation, alternative splicing, RNA transport, local translation, stress granules formation, and more recently, DNA damage [83,84]. FUS is a heterogeneous nuclear ribonucleoprotein (hnRNP) and a member of the FET (FUS/EWS/TAF15) protein family. In the context of miRNA processing, FUS was shown to bind specific neuronal pri-miRNAs at their terminal loop sequences and to enhance their processing [62]. Using chromatin immunoprecipitation (ChIP) experiments, Morlando *et al.* [62] demonstrated that FUS is recruited to the transcription sites of specific miRNAs in a RNA-dependent manner and mediates the co-transcriptional recruitment of Drosha at the same sites. Accordingly, Drosha recruitment at these specific miRNA loci is lost upon FUS depletion. Interestingly, the miRNAs that were analyzed (miR-9, miR-125b and miR-132) have relevant roles in crucial neuronal functions, such as synaptogenesis and differentiation [39,53]. Recently, these observations were partially confirmed and extended by the identification of a regulatory loop in which FUS participates in miRNA biogenesis and is in turn regulated by miRNAs [63]. In the context of this review, it should be mentioned that FUS mutations have been found in patients affected by Amyotrophic Lateral Sclerosis (ALS), and Fronto-Temporal Lobar Degeneration (FTLD), two related yet distinct neurodegenerative disorders, and more recently in Essential Tremor (ET) [85,86,87]. ALS is a neurodegenerative disorder that affects both upper and lower motor neurons eventually leading to paralysis. Most ALS patients die within three to five years after symptom onset. A majority (about 90%) of ALS cases are sporadic in nature with an unknown etiology, while the remaining 10% of cases are attributed to inheritable genetic defects (for extensive reviews see [88,89]). FTLD is characterized by progressive decline in behavior, personality, or language, symptoms that are attributed to the degeneration of the frontal and temporal lobes [90]. Finally, ET is the most common tremor disorder. The disease incidence and prevalence increase with age, such that as many as 22%–23% of people aged more than 90 years have ET (reviewed in [91]). Interestingly, an ALS-linked mutation in the 3'-UTR of FUS mRNA impairs the binding of miR-200 and miR-141 increasing FUS expression. This in turn stimulates miR-200 and miR-141 processing. In this circuitry, is also involved Zeb1, a target and a transcriptional repressor of miR-141 and miR-200a [63].

Also, the other two members of the FET family have also been involved in ALS and FTLD disorders [92,93,94] and were identified as Drosha interacting proteins [95,96]. In addition, TAF15 (TAF15 RNA polymerase II, TATA box binding protein (TBP)-associated factor, 68 kDa) has been shown to affect miR-17 biogenesis probably by acting at the transcriptional level [97]. In contrast, EWS (EWS RNA-binding protein 1, EWSR1) appears to indirectly influence pri-miRNA processing by acting as a negative regulator of Drosha transcription [98].

#### 2.1.2. Modulation of Drosha RNAse III Activity

Drosha cleavage activity is regulated by several RBPs. Although in most cases their action is still poorly characterized at the molecular level, two mechanisms can be envisaged. RBPs can either facilitate Drosha positioning and cropping by relaxing pri-miRNA structures, or they can inhibit processing by sterically hindering Drosha cleavage sites. Below, we will summarize recent work that has uncovered a role in the modulation of Drosha activity for RBPs that are linked to physiological and pathological neuronal functions.

TDP-43 (TAR DNA-binding protein-43, TARDBP) is a predominantly nuclear DNA/RNA-binding protein, which regulates mRNA biogenesis at several steps and can positively regulate Drosha activity. TDP-43 has been implicated in transcription regulation, mRNA alternative splicing, transport and translation. Furthermore, TDP-43 was primarily identified as a component of the cytoplasmic aggregates that are deposited in the neurons of the affected tissues of ALS and FTLD, suggesting that a loss of its nuclear functions may contribute to the pathology (for a complete review see [99,100,101]). In addition, TDP-43 is known to interact with the Drosha complex [60,96,102] and to bind specific pri-miRNAs [60]. The Drosha–TDP-43 interaction is partially mediated by RNA and was shown to increase the affinity of Drosha for specific pri-miRNA, thus enhancing their processing [60]. Pri-miR-132, a well-known player in neuronal outgrowth [39,58], has been identified as one of TDP-43 targets [60]. Upon siRNA-mediated TDP-43 depletion in differentiating Neuro2a cells, miR-132 processing is partially inhibited and neuronal outgrowth is attenuated; the phenotype was partially recovered by overexpression of pri-miR-132, thus further supporting a physiological role for TDP-43 in neuronal differentiation [60]. Furthermore, TDP-43 has also been involved in the regulation of Drosha expression by a mechanism that is still not completely understood [102] and that will be discussed below.

In contrast to TDP-43, Lin28 provides an example of negative modulation of Drosha activity. The RBP Lin28 and its miRNA target let-7 are important players in stem cell differentiation and in development (reviewed in [66,103]). Lin28 and let-7 form a regulatory circuit that is developmentally regulated: while Lin28 represses the expression of let-7 [67,71,104], let-7 downregulates Lin28 [67]. The regulation of let-7 by Lin28 is required for normal development and contributes to pluripotency by preventing let-7-mediated differentiation of embryonic stem cells [67,71]. Let-7 expression appears particularly relevant in neuronal cells; both during brain development and differentiation of neuronal stem cells let-7 levels are upregulated [67,105].

While *C. elegans* has a single Lin28 gene, the mammalian genome encodes two Lin28 paralogues, Lin28 (also named Lin28A) and Lin28B [106,107,108,109]. The two Lin28 paralogues display different subcellular localizations and different mechanisms of let-7 downregulation. Lin28A localizes mainly to the cytoplasm where it stimulates degradation of pre-let-7 (see below in the pre-miRNA processing paragraph). Lin28B instead predominantly localizes to the nucleoli [72], where it sequesters pri-let-7 away from the Microprocessor thus preventing its processing [74].

NF45 (90 kDa interleukin enhancer-binding factor 2, ILF2) and NF90 (90 kDa interleukin enhancer-binding factor 3, ILF3) are other two RBPs that negatively regulate pri-let 7 processing. Both proteins have been identified as Drosha-interacting proteins [96]. NF45 and NF90 are dsRNA-binding proteins that form a heterocomplex. The NF45/NF90 heterodimer binds the primary let-7 transcript negatively affecting its processing probably by reducing Microprocessor accessibility [74]. However, this inhibition seems to be nonspecific since other miRNAs (pri-miR 15a/16-1, pri-miR-21 and pri-miR-30a) were similarly inhibited [74].

MSI2 (musashi RNA-binding protein 2) and HuR (ELAV-like RNA binding protein 1, ELAVL1) are two RBPs that were originally associated with the cytoplasmic polyadenylation of mRNAs (for review see [110]) but were also shown to negatively regulate pri-miR-7 processing [111]. Specifically, HuR-mediated MSI2 binding to pri-miR-7 strongly impairs its processing by Drosha, by increasing the rigidity of the pri-miR-7-1 stem-loop structure [111]. Consistently, upon differentiation, neuroblastoma cells display a reduction of HuR and MSI2 levels as well as an increment in mature miR-7 level without changes of the primary miR-7 level confirming a post-transcriptional regulatory mechanism [111]. Interestingly, miR-7 has been identified as a modulator of alpha synuclein expression [78]. Alpha synuclein forms aggregates in the dopaminergic neurons of Parkinson’ disease (PD) patients [112,113]. Accordingly, in pharmacological models of PD, both in cultured cells and in mice, miR-7 is downregulated and α-synuclein protein levels increased, suggesting an involvement of miR-7 in PD pathogenesis [78]. Although an involvement of MSI and HuR proteins in PD pathology has not yet been reported, a possible role cannot be excluded.

Finally, hnRNP A1 (heterogeneous nuclear ribonucleoprotein A1) provides an example of an RBP that can act in opposite ways on miRNA processing depending on the specific miRNA. HnRNP A1 is a nucleocytoplasmic shuttling protein with many roles in RNA metabolism. HnRNP A1 protein levels were found drastically reduced in protein extracts and entorhinal cortical sections of Alzheimer’s disease (AD) patients [114]. In mice, the specific depletion of hnRNP A1 in the entorhinal cortex results in reduced learning and memory capabilities, supporting a functional role of hnRNP A1 in cortex-related functions [114] (for a review about hnRNP-A1 and neuronal functions see [115]). HnRNP-A1 binds different pri-miRNAs affecting their processing by Drosha in a opposite manner [76,77]. In the case of miR-18a, hnRNAP-A1 facilitates Drosha cleavage by binding to the pri-miRNA and inducing a relaxation of the secondary structure [77]. In the case of pri-let-7, instead, hnRNP-A1 binding to the conserved stem inhibits its cleavage by Drosha. In this case, hnRNP-A1 competes with KSRP (KH-type splicing regulatory protein, KHSRP) for pri-let-7a-1 binding, which in contrast acts as a positive regulator of the Microprocessor complex [76,116].

#### 2.1.3. Regulation of Drosha Protein Expression

While modulation of Drosha enzymatic activity by RBPs can affect processing of specific miRNAs, changes of Drosha expression level are expected to generally influence global miRNA expression. One notable example of regulation of Drosha expression is provided by the action of DGCR8, its partner in the Microprocessor complex. DGCR8 was shown to stabilize Drosha through protein-protein interactions [117]. Interestingly Drosha also affects DGCR8 expression by cleaving DGCR8 mRNA. This cross-regulatory loop maintains homeostatic levels of Microprocessor activity [117].

The FET family member EWS has been involved in the regulation of Drosha expression, although its function is controversial and may be tissue-specific. In EWS null mice, Drosha is downregulated in brain and lungs extracts [48,95]. However, in primary fibroblasts depleted of EWS, Drosha appears to be upregulated [98]. The characterization of protein-DNA occupancy revealed an enrichment of EWS at the Drosha promoter. Moreover, *in vitro* and *in vivo* experiments demonstrated that Drosha transcription is downregulated in a EWS dependent manner [98] and suggest that EWS acts on the Drosha promoter most likely as a transcriptional repressor [98].

Finally, TDP-43, in addition to the effect on Drosha enzymatic activity described above, was also shown to modulate Drosha protein levels. During *in vitro* neuronal differentiation, TDP-43 depletion results in a reduction of Drosha protein levels and in a global down-regulation of miRNA expression [102]. Although the exact mechanism remains to be explored, the authors suggest a TDP-43 involvement in proteasome-mediated Drosha degradation [102].

### 2.2. Pre-miRNA Processing

Similarly to Drosha, also Dicer-mediated pre-miRNA processing can be regulated by RBPs (Figure 1B). Two main mechanisms can be envisaged: (i) direct RBP interaction with pre-miRNAs either improving or impairing their cleavage by Dicer, and (ii) regulation of either expression level or localization of Dicer.

#### 2.2.1. Pre-miRNA Export and Further Cleavage by Dicer

The pre-miRNAs that are generated by Drosha in the nucleus are then exported into the cytoplasm, where maturation is completed. In neurons, the presence of specific structures, such as dendrites and axons, represents an additional layer of complexity, because pre-miRNA processing can be completed locally upon specific stimulus.

MiR-134 has been shown to be important for synapse development and function [118,119]. Not surprising, pre-miR-134 displays a specific localization: it is enriched in dendrites of primary hippocampal neurons and at synapsis *in vivo* [59,118]. It has been reported that miR-134 negatively regulates the dendritic spine sizes by targeting kinase LimK1 (Lim-domain-containing protein kinase 1), hence affecting the synaptic function [118]. In a recent work, Birker and colleagues found a dendritic targeting signal located in the pre-miR-134 terminal loop [59]. By using pull down experiments, the authors identified the RBP DHX36 (DEAH (Asp-Glu-Ala-His) box polypeptide 36) as the major protein interacting with the pre-miR-134 terminal loop [59]. As a consequence of this interaction, two main effects were observed: inhibition of Dicer-mediated cleavage, and localization of pre-miR-134 in dendrites [59]. Accordingly, upon DHX36 depletion, dendritic pre-miR-134 localization is significantly lost, LimK1 is upregulated and dendritic spines are enlarged [59]. *In vitro* DHX36 competes with Dicer for pre-miR binding [59]. On the basis of these observations, the authors propose a model in which DHX36 drives pre-miR-134 to dendrites and keeps it inaccessible to Dicer until neuronal activity promotes the release, allowing maturation [59].

TDP-43 has been also involved in the pre-miRNA processing regulation, thus participating in almost all steps of miRNA biogenesis. TDP-43 binds specific terminal loops of pre-miRNAs, in particular pre-miR-143 and pre-miR-574, in the cytoplasm and interacts with Dicer and Ago2 in a partial RNA dependent manner. Upon TDP-43 depletion, the processing of the pre-miRNAs mentioned was selectively impaired, thus indicating a positive role of TDP-43 in the processing of those pre-miRNAs [60].

Lin28A provides a peculiar mechanism of regulation of pre-miRNA processing. Lin28A binding to the conserved terminal loop of pre-let-7 induces its 3' terminal uridylation through the recruitment of a non-canonical poly (A) polymerase, TUTase4 (TUT4). This modification blocks Dicer-mediated processing leading to degradation [72,73,120].

#### 2.2.2. Regulation of Dicer Expression or Localization

AUF1 (AU-rich element RNA binding protein 1, also known as hnRNPD) regulates the stability of several transcripts including DICER1 mRNA [121]. AUF1 interacts with both the coding region and the 3'UTR of DICER1 mRNA, decreasing mRNA stability. As expected, upon AUF1 expression a global effect of the AUF1-mediated reduction of Dicer is observed, leading to a decrease in the abundance of the 20 miRNA tested [121]. The neurological effects of Dicer depletion have been characterized in several models and encompass from brain malformations to defects in neuronal functions (for a review see [122]).

### 2.3. RISC Loading

The final step in miRNA biogenesis consists in the loading of the small RNA duplex generated by Dicer onto the RISC, and the removal of the passenger strand to generate a mature RISC (Figure 1C). RBPs can also act at this level to further modulate miRNA biogenesis. One example is provided by the ubiquitin-ligase TRIM32 (tripartite motif containing 32), which has been shown to regulate let-7 production at the level of RISC. In a first study, Schwamborn *et al.* demonstrated that TRIM32 enhances let-7 activity by acting as RISC cofactor promoting neuronal differentiation [105]. In a more recent paper, TRIM32 is shown to participate with the RNA helicase DDX6 (DEAD (Asp-Glu-Ala-Asp) box helicase 6) and the RISC component Ago2 in let-7-mediated neuronal differentiation [75]. Although the precise mechanism needs to be further explored, it is clear that the cooperative interaction of these proteins at the level of RISC positively affect let-7 activity.

TDP-43 provided once more an example since it was shown to negatively affect the loading of miR-1 and miR-206 onto the RISC complex [123]. TDP-43 depletion increases both miR-1 and miR-206 interactions with Ago2 in muscle cultured cells and stimulates both miRNAs activities as measured by using a specific luciferase assay reporter *in vitro* [123]. Furthermore, *Drosophila* mutants in the ortholog of TDP-43, TBPH, display enhanced activity of miR-1 [123]. Consistent with a negative role of TDP-43 in RISC loading, mice over-expressing wild-type TDP-43 show increased expression of IGF-1 (insulin-like growth factor 1) and HDAC4 proteins, two known targets of miR-1 and miR-206, even if both mature miR-1 and miR-206 are highly expressed [123].

## 3. RBPs Regulate miRNA Function

RBPs could affect not only miRNAs biogenesis but also their function by facilitating or counteracting the regulation of the target mRNA. Here, several examples both of cooperation and competition mechanisms of regulation are reported (Table 2).

**Table 2:** RBPs regulating miRNA function.

RBPs	miRNA	Common mRNA Target	Mechanism	References *
FMRP	miR-125b	NR2A	cooperation	[39]
FMRP	miR-125a	PSD-95	cooperation	[124]
TDP-43	miR-NID1	NRXN1	cooperation	[125]
HuR	miR-494	NCL	competition	[126]
HuD	miR-129	Kv1.1	competition	[127]
hnRNP L	miR-297, miR-299	VEGFA	competition	[128]

* articles where RBP-miRNA regulation was reported.

### 3.1. Cooperative Regulation between miRNAs and RBPs

**Figure 2:**
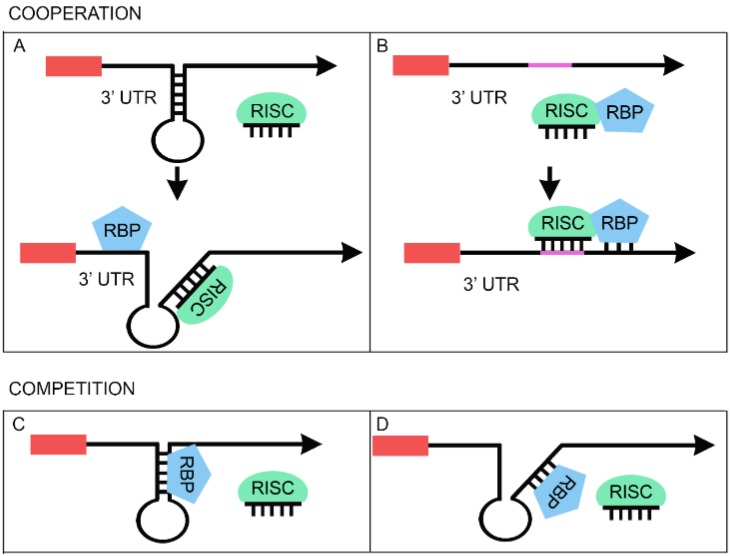
Functional interaction between microRNAs and RBPs. Upper panel: cooperative model. The RBP can enhance the miRNA effect on the common mRNA target by opening the RNA secondary structure (**A**). Alternatively, the RBP can improve the transport of the miRNA loaded in the RISC complex to its target (**B**). Lower panel: competitive model. Competition could take place when the binding of RBP on the mRNA prevents the base-pairing of the miRNA by changing the secondary structure of the mRNA (**C**). Alternatively, an antagonistic interaction occurs when the RBP competes with miRNA for the binding to the target site and thus reduces the silencing effect of the miRNA (**D**).

An increasing number of observations, mostly derived from cancer-related studies (for a recent review see [129]), show that the main players of cell proliferation could be controlled by the synergic effect of RBP and miRNAs. This effect may be due to a cooperative interaction in which the RBP facilitates miRNA binding to the 3'UTR of the shared target mRNA in either of two ways. First, the RBP can make the miRNA seed sequence in the 3'UTR more accessible through the opening of the RNA secondary structure (Figure 2A). Alternatively, the RBP can enhance the transport of the miRNA loaded in the RISC complex to its target (Figure 2B). The clearest example of the first model is represented by the Pumilio proteins, PUM1 and PUM2, that cooperate with several miRNAs by opening the secondary structure of their target transcripts (for review see [129,130,131]). A similar mode of action most likely applies to the case of FMRP (Fragile X mental retardation protein), one of the first RBP that was shown to associate with RISC in *Drosophila* [131], suggesting a potential role in its modulation of miRNAs function. The FMRP protein, most commonly found in the brain, is essential for normal cognitive development. Its absence (due to silencing of the FMR1 gene) causes fragile X syndrome, an X-linked neurodevelopmental disorder (for review see [132]). A specific mutation that is linked to Fragile X syndrome was shown to affect FMRP association with RISC [131]. Further work in the last five years confirmed this association and revealed that FMRP cooperates with several miRNAs in the silencing of specific mRNAs, thereby modulating the structure and the function of synapses [39,132]. In particular, Edbauer and colleagues demonstrated that FMRP binds miR-125b and miR-132 in mouse brain and that the overexpression of both miRNAs modifies dendritic spine morphology. The effects induced by the expression of both miRNAs are abolished by FMRP knock-down [39]. Edbauer and colleagues identified and validated NR2A, a subunit of NMDA receptors, as a target of miR-125b [39]. Moreover, they found that the NR2A mRNA immunoprecipitated with FMRP. Importantly, deletion of the major miR-125b target site within the 3'UTR impairs the upregulation of NR2A that is observed in FMRP knock-down cells [39]. Based on these data, the authors propose that a functional interaction between FMRP and miR-125b is crucial for the control of NR2A expression.

A similar cooperative regulation was recently reported for the PSD-95 (postsynaptic density protein 95) mRNA, a key player in the control of AMPAR endocytosis, synaptic strength and spine stabilization [124,133]. Initially, Muddashetty *et al.* [124] validated PSD-95 as a target of mir-125a and demonstrated that this miRNA was essential for mGluR (metabotropic Glutamate Receptor)-mediated translation in neurons. Then, they characterized the mechanism by which FMRP regulates mGluR-mediated translation of PSD-95 mRNA [124,134]. Essentially, they found that both FMRP and miR-125a are fundamental for mGluR-mediated regulation of PSD-95 mRNA translation at the synapse. FMRP apparently controls the execution of miR-125a-mediated silencing of PSD-95 mRNA, since it is necessary for the recruitment of miR-125a-AGO2 complexes on the 3'UTR of PSD-95 mRNA [124]. Moreover, they showed that the phosphorylation status of a specific serine residue (Ser 499) in FMRP is the critical switch required for relieving miR-mediated inhibition of PSD-95 mRNA translation. While the phosphorylated form of FMRP preferentially formed the inhibitory complex with miR-125a, stimulation of mGluRs led to the dephosphorylation on FMRP by the specific phosphatase PP2A [124].

A potential example of the second mode of regulatory cooperation between an RBP and a miRNA (Figure 2B) is proposed in a recent paper from the Chen group [125]. In this study, the authors analyzed recently published CLIP-seq data for TDP-43 from human and mouse samples [135] and identified binding to pre-miRNAs, miRNAs, piwiRNAs and mRNAs. They also identified a novel human intronic miRNA, miR-NID1 (for NRXN1 intron-derived miRNA, which corresponds to miR-8485), which appears to regulate the expression of its host gene, neurexin 1 (NRXN1). Neurexins are proteins that function as cell adhesion molecules and receptors in synaptogenesis. In this work, the authors demonstrated that TDP-43 directly binds miR-NID1 [125]. Moreover, when either TDP-43 or miR-NID1 was depleted, the level of NRXN1 was significantly increased indicating that TDP-43 is involved in miR-NID-mediated NRXN1 silencing [125]. However, how TDP-43 and miR-NID1 act is still unclear. While no target sites of miR-NID1 could be found in NRXN1 mRNA, a potential target was found in the promoter region of NRXN1 by bioinformatic prediction [125]. Based on this observation, the authors propose a model in which TDP-43 would enhance the import of miR-NID1 into the nucleus where it would act in the transcriptional silencing of the NRXN1 gene [125]. Since there is no evidence to support this model, more experiments are needed to understand the relationship between TDP-43 and miR-NID1.

### 3.2. Competitive Regulation between miRNAs and RBPs

In addition to evidence indicating that miRNAs and RBPs work synergistically to silence protein expression, there are also reports of competition between RBPs and miRNAs that can exert opposing effects on target’s expression. Notably, the competition seems to be a more complex scenario considering that the fate of the specific mRNA is the result of a dynamic balance between two (or more) opposite forces. Several studies have provided some key details that suggest two likely models that explain how RBPs could contrast miRNAs’ function. On one hand, the RBP and the miRNA can compete for the same binding site (e.g., by steric hindrance) (Figure 2D). On the other hand, the interaction of the RBP with the mRNA can prevent miRNA binding to a distinct part of the transcript (e.g., by changing mRNA secondary structure) (Figure 2C).

Competition for the binding site appears to be the prevalent mode of action of the proteins of the Hu/ELAV family, which mainly stabilize target mRNAs. The most recent study describing the competitive role of HuR showed evidence that this RBP associates with the 3'UTR of the nucleolin (NLC) mRNA. This interaction promotes the initiation of NLC translation without affecting NCL mRNA half-life [126]. Further insight into the regulation of NCL expression came from the identification of a miRNA, miR-494, which lowers NCL expression. Importantly, Tominaga and colleagues observed that HuR binding to NCL mRNA was strongly reduced when miR-494 was overexpressed, suggesting that miR-494 competes with HuR, displacing it from the NLC mRNA [126]. Instead, HuR silencing increased the interaction of Ago with NCL mRNA, suggesting that HuR might prevent the miRNA-RISC-induced silencing effect. Based on these results, the authors propose that HuR competes with miR-494 for the regulation of NCL production: while HuR prevents the recruitment of NLC mRNA to P-bodies, miR-494 favors it [126]. The result of the competing actions of HuR and miR-494 determines the efficiency of NCL translation. The regulation of NCL synthesis is a crucial step for several diseases. Its level is significantly elevated in many cancers and a pathogenic role in neurodegeneration has been clearly reported too. Interestingly, NCL was shown to be downregulated in SN (substantia nigra) tissues from Parkinson subjects and in a dopaminergic neuron cell line, upon treatment with rotenone [136]. Moreover, NCL overexpression is reported to be neuroprotective and its downregulation promotes neuronal death in dopaminergic cells [136], and in various models of polyglutaminopathies [137,138]. However, HuR is also known to act in a cooperative manner with miRNA to facilitate target silencing [138]. The neuronal family member of the Hu proteins, HuD, can act in a similar manner on miRNA-129. Sosanya and colleagues showed that inactivation of the *mTORC1* (mammalian target of rapamycin complex 1) kinase by rapamycin treatment increases the affinity of HuD for *Kv1.1* (Potassium voltage-gated channel subfamily A member 1) mRNA coding region and enhancing its translation in dendrites [127]. Notably, the Kv1.1 3'UTR contains a conserved binding site for miR-129. Using an RNA affinity capture system, Sosanya and colleagues found that this miRNA binds to Kv1.1 mRNA when mTORC1 kinase is active, while HuD binds other mRNA targets, such as *CaMKII*α (Ca^2+^/calmodulin-dependent protein kinase II) [127]. Moreover, the authors showed that upon inactivation of mTORC1, binding of the HuD protein to Kv1.1 overcomes miR-129 repression of Kv1.1 mRNA. Similarly, the overexpression of HuD relieves miR-129-mediated silencing when mTORC is active [127]. This is one of the best examples to date for the mechanism proposed by W. Filipowicz [139]. This model suggests that RBPs can displace miRNAs-induced silencing complexes from target mRNA even when its binding site and the miRNA site are not located in close proximity [140,141].

The interplay between miRNAs and RBPs is also relevant in stress conditions due to exogenous stimuli. This is highlighted by a recent study on the post-transcriptional regulation of vascular endothelial growth factor A (VEGFA) in hypoxic condition. Jafarifar and colleagues found that miR-297 and miR-299 bind the CA-rich element (CARE) in the 3'UTR of VEGFA silencing its expression [128]. Upon hypoxia VEGFA level increases. The authors found that this effect is not due to an inhibition of the miRNAs’ expression but rather to the stabilization of the VEGFA mRNA mediated by hnRNP L that binds to CARE in hypoxic conditions [142]. HnRNP L is a nucleo-cytoplasmic shuttling protein that is normally localized to the nucleus but that relocalizes to the cytosol in hypoxic conditions [128]. In the cytoplasm, hnRNP L competes with the miRNA for VEGFA mRNA binding. Indeed, the authors show that cotransfection of hnRNP L together with one of the CARE-binding miRNAs can restore the expression of the endogenous VEGFA [128]. Significantly, this study highlights the importance of regulation of the subcellular localization of RBPs for their function. These results were obtained in monocyte cells, and for the purpose of this review it is important to point out that the circulating monocytes are precursors of the microglia, which are involved in the pathogenesis of ALS. In a recent paper, Moreau and co-workers demonstrated clinical and functional abnormalities in the HIF-1 (Hypoxia-inducible factor) pathway and an associated decrease in VEGF production during hypoxia in monocytes from sporadic ALS patients [143]. However, the mechanism underlying these abnormalities is still unclear. Thus, it would be interesting to investigate the regulation achieved by RBPs and miRNAs in VEGFA mRNA in ALS.

## 4. Perspectives and Conclusions

In recent years, we have discovered that a large fraction of the cellular transcriptome, far from representing “rumor” of the transcription machineries, may instead be involved in the careful fine-tuning of crucial cellular functions. This is particularly relevant in the nervous system where complexity is the rule. Neuronal plasticity requires the reinforcement as well as the formation of specific connections. All these functions should be carefully regulated. In this context, the phenotypic effects of Dicer ablation in different model organisms clearly indicate that miRNAs play an important role in neurogenesis as well as in neurodegeneration. However, the precise contribution of specific miRNAs to neuronal homeostasis is far from being understood. Another open question concerns the contribution of the RBP-miRNA regulatory circuitry to the physiology of neurons. Since many of these proteins have additional functions in the processing, translation and decay of messenger RNA, the relative contribution of each function is difficult to determine. Moreover, although the molecular details of the regulatory role of RBPs in the processing and/or function of specific miRNAs are progressively emerging, to what extent alterations in these events are directly linked to the disease is still unclear. Another interesting issue concerns the limitations of the experimental models used for the investigation of neurodegenerative processes. Studies aiming at elucidating the molecular events underlying various human neurodegenerative disorders have utilized patients’ post-mortem tissues and transgenic animal models. Whereas post-mortem tissues are not always available and often represent the end stage of the disease, the animal models do not fully recapitulate the human disease phenotype. Thus, the development of reprogramming techniques that offer the possibility to obtain neurons from somatic cells of patients in previously unachievable amount and quality represents an exciting scenario for elucidating the etiology of neurodegenerative diseases and for the development of potential miRNA-based therapeutics [144,145].
